# Mechanical property dependence on compositional heterogeneity in Co-P metallic nanoglasses

**DOI:** 10.1038/s41598-024-58247-9

**Published:** 2024-03-29

**Authors:** Tian Li, Nana Li, Shengming Zhang, Guangping Zheng

**Affiliations:** 1 CDGM Glass Co., Ltd., Chengdu, 610199 China; 2 Chengdu Guangming Paite Precious Metal Co., Ltd., Chengdu, 610199 China; 3 Department of Mechanical Engineering, The Hong Kong Polytechnic University, Hung Hom, Kowloon, 999077 Hong Kong SAR China; 4 The Hong Kong University of Science and Technology, Clear Water Bay, Kowloon, 999077 Hong Kong SAR China

**Keywords:** Metallic nanoglasses, Glass–glass interfaces, Plastic deformation, Molecular dynamics, Nanoindentation, Materials science, Nanoscience and technology

## Abstract

The glass–glass interfaces (GGIs) are in a unique glass phase, while current knowledge on the interfacial phase has not completely established to explain the unprecedented improvements in the ductility of metallic nanoglasses (NGs). In this work, Co–P NGs prepared through the pulse electrodeposition are investigated, whose GGI regions clearly show elemental segregation with chemical composition dominated by element Co. Such compositional heterogeneity is further verified by molecular dynamics (MD) simulation on the formation of GGIs in Co-P NGs and atomic structures of GGIs with Co segregation are found to be less dense than those of glassy grains. More importantly, Co segregation at GGIs is closely related to the improved ductility observed in Co-P NGs, as demonstrated by nanoindentation measurements and MD simulations. This work facilitates the understanding on the relations between compositional heterogeneity and improved ductility as observed in Co-P NGs, and thus opens a new window for controlling the mechanical properties of NGs through GGI engineering.

## Introduction

In 1989, a new type of noncrystalline alloys-the metallic nanoglasses (NGs)-were firstly proposed^1^. Basically, NGs are heterogeneous in nanostructures that compose of glassy grains interconnected by planar defects called glass–glass interfaces (GGIs)^2–7^. The atomic^8–12^ and electronic^13,14^ structures of GGIs much differ from those in the interiors of glassy grains, which could be well described by a classical dual-phase model for those of NGs. For instance, Fang et al.^11^ studied GGIs by using positron annihilation spectroscopy and the GGIs were found to be less dense. An additional ferromagnetic component was determined at GGIs by Mossbauer spectroscopy analysis on NGs containing non-ferromagnetic glassy grains^14^, indicating that there could be significant contribution of itinerant electrons in the GGI regions. In general, the thermodynamic^15–19^, magnetic^20–27^, and mechanical^28–36^ properties of NGs are much altered by the GGIs.

Besides some interesting physical and chemical properties that are previously discovered in NGs^25,26,37^, e.g., catalysis, the mechanical properties of NGs have attracted attention over the last decade. It has been found that the tensile ductility of NGs could be much improved by the introduction of GGIs. The molecular dynamics (MD) simulations^8,38–41^ suggested that the NGs possessed multiple shear bands under the plastic deformation and would deform continuously without exhibiting a sudden stress drop on the tensile curves. Wang et al.^28^ determined the mechanical properties of NGs through a microscale tensile test, and they found that NGs could exhibit an overall engineering strain up to 15%. In other words, GGIs may enable glassy alloys with decent ductility although the mechanisms are still unclear. Such lack of understanding on GGI-controlled plastic deformation is mainly attributed to the difficulties in atomistic modeling of multicomponent amorphous alloys and to the limitations of current experimental tools, which are critical to revealing the dependence of ductility on the atomic structures and thermodynamic properties of GGIs.

Recently, compositional heterogeneity resulting from the elemental segregation at GGIs is reported^42–45^. Taking Pd-Si NGs as an example^42^, an enrichment of Pd elements and a depletion of Si atoms were characterized for the GGIs, in comparison with those in the interiors of glassy grains. Similarly, Fe segregation at the GGI regions was observed in Fe-Sc NGs^45^. Nonetheless, there is a lack of experimental and simulation results for a consistent NG system, which is necessary to correlate this phenomenon with the improved ductility as observed in NGs. The previous simulation studies mainly focus on Cu-Zr NGs^31,44^ and are yet not available for many other NG systems. In our previous work^15,25,27,46–48^, it was demonstrated by us that the pulse electrodeposition is a feasible synthesis route in preparing (Co, P)-based NGs, which could be further investigated by the MD simulation^49^. In this work, the mechanical property dependence on compositional heterogeneity in Co-P NGs prepared through pulse electrodeposition has been investigated. The experimental and simulation studies on Co-P NGs suggest that the segregation of Co atoms to the GGI regions would lead to the formation of a *new glass phase* in the GGI regions. Furthermore, the atomic structures of GGIs with an enhanced Co segregation could promote the shear banding in NGs under plastic deformation, demonstrating that the improved ductility is closely related to the compositional heterogeneity in NGs. The results show that it is feasible to develop a novel strategy in improving the ductility of NGs through controlling the composition of GGI phases.

## Methodologies

### Experimental methods

Co-P NGs with a thickness of 0.05–0.1 mm were fabricated through pulse electrodeposition in a three-electrode electrochemical cell. The NG films were deposited on a titanium substrate, which was the working electrode rotating at 1000 rpm. The platinum foil and saturated calomel electrode were used as counter electrode and reference electrode, respectively. The composition of the electrolytes was described as follows: CoSO_4_·7H_2_O (0.1 mol/L), C_6_H_5_Na_3_O_7_·2H_2_O (0.2 mol/L), H_3_BO_3_ (0.5 mol/L), and NaH_2_PO_2_·H_2_O (0.2 mol/L). The PH value of the electrolyte was adjusted to 2.0–3.0 by adding concentrated H_2_SO_4_. The growth of glassy grains could be well restricted through high working potential, low duty cycle and high pulse frequency. Thus, a signal generator was utilized to supply pulse voltage signals with a duty cycle of 30% at 100 kHz to potentiostat. A working potential of 3.8 V was maintained at each duty cycle throughout the plating processes. Teflon mold was used to prepare the NG films in a rectangular shape (30 mm × 3.5 mm), which were mechanically exfoliated from titanium substrates as free-standing films for further experimental studies.

The amorphous state of NG films was characterized by X-ray diffraction (XRD, Rigaku Smartlab). The scanning electron microscope (SEM, Tescan Vega 3) was used to observe the nanostructures and chemical composition was evaluated by the atom probe tomography (APT, LEAP 5000HR). High-resolution images of glassy grains with sizes less than 50 nm in diameter were taken by the transmission electron microscopy (TEM, JOEL JEM-2011). Thermodynamic properties were measured by the differential scanning calorimetry (DSC, TA Instruments Q200) and dynamic mechanical analysis (DMA, TA Instruments Q800). For the DMA experiments, NG films were mounted on the 20-mm dual cantilever clamps and were forced to oscillate with an amplitude of 20 μm. Nanoindentation tests (Bruker Hysitron TI980) were performed on 0.1 mm-thick NG films by using a Berkovich indenter. Before nanoindentation measurements, NG films were firmly bonded to a sample holder by superglue. At least 100 indentation sites were selected for each applied load (chosen to be 2–10 mN) to measure the reduced modulus and hardness. For creep experiments, at least 10 indentation events were conducted for each applied load (chosen to be 5–20 mN), which was held for 200 s.

### Simulation methods

The MD simulations were performed using large-scale atomic/molecular massively parallel simulator^50^. The interatomic potentials between Co–Co, Co-P and P-P were described by the Pak–Doyama type pair potential^51^, with the potential energy (E_ij_) in eV between atoms i and j written as follows:

1 $${\text{E}}_{{{\text{ij}}}} \left( {\text{r}} \right) = {\text{a}}\;\left( {{\text{r}} - {\text{b}}} \right)^{4} + {\text{c}}\,\left( {{\text{r}} - {\text{d}}} \right)^{2} + {\text{e}},\;\;\;{\text{r}} < {\text{r}}_{0} ,$$

where r was the distance between atom i and atom j. E_ij_ would be set to 0 eV when r was greater than the cutoff radius. Table 1 summarizes the parameters of Pak-Doyama type pair potentials in detail. It should be worth noting that the amorphous atomic structures of Co-P glasses in MD simulations are in good agreements with those observed in the experiments^49^, demonstrating the effectiveness of the interatomic potentials used for simulating the mechanical properties of Co-P NGs. The equations of motion were numerically integrated at a time step of 1 fs, and the periodic boundary conditions were applied in x, y, and z directions. Barostat and Nose–Hoover thermostat were utilized to control the pressure and temperature of simulation model systems, respectively.

**Table 1:** The parameters of Pak-Doyama type pair potentials for Co-P amorphous alloys.

E_ij_ (eV)	a	b	c	d	e	r_0_ (Å)
E_Co-Co_	− 0.12812	1.82709	1.15421	2.50849	− 0.13448	3.34
E_Co-P_	− 0.15374	1.58709	1.38505	2.26849	− 0.13167	3.20
E_P-P_	− 0.07435	2.60709	0.64791	3.27885	− 0.07531	4.21

**Figure 1:**
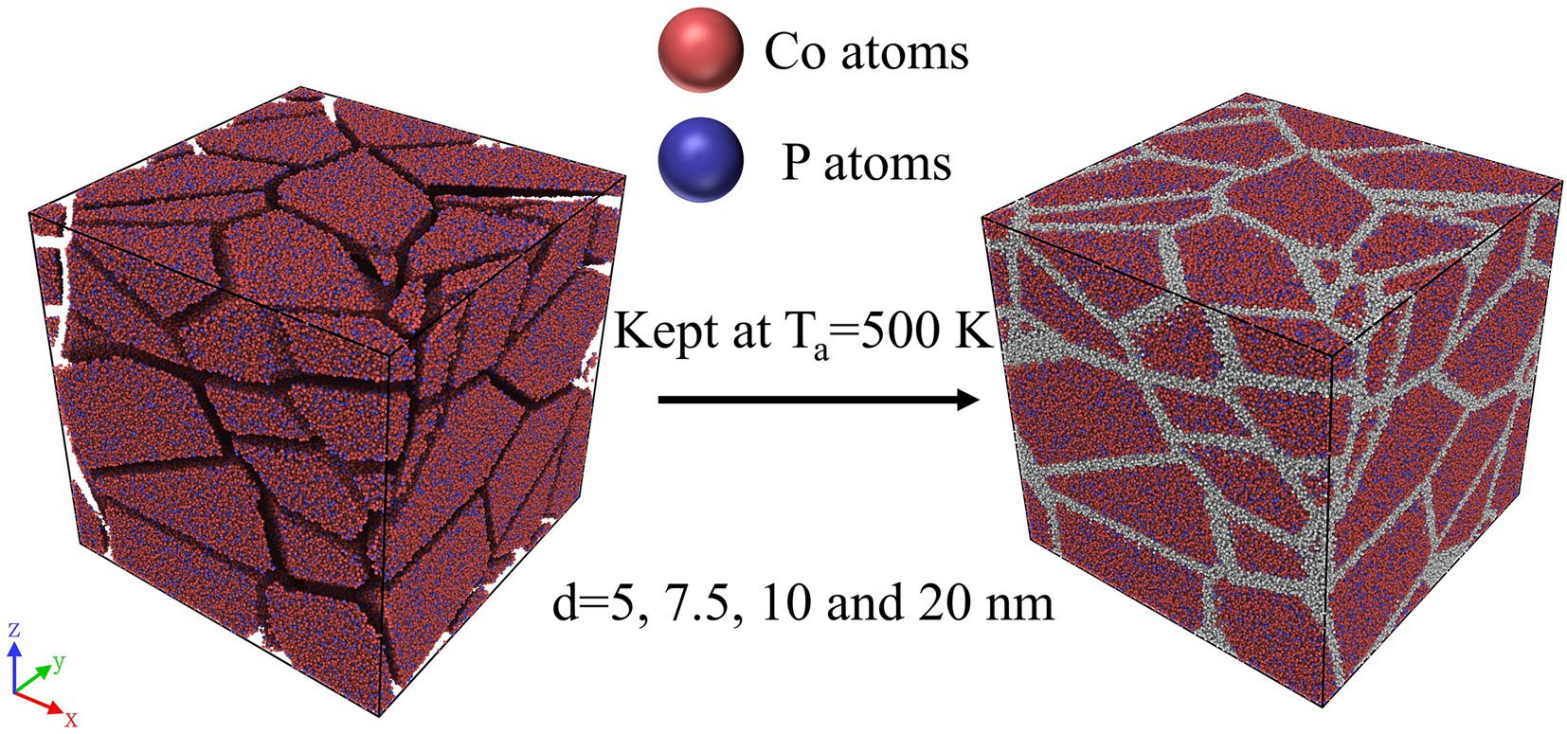
Schematics on the formation of Co-P NGs in the MD simulations, where GGI atoms are highlighted with grey color.

A crystalline Co_80_P_20_ alloy was initially constructed^52^ and relaxed at 300 K under isobaric–isothermal ensemble. The relaxed crystalline Co_80_P_20_ alloy was first kept at 2000 K until it was completely transformed into the liquid phase. The melts were then rapidly quenched to 300 K at a cooling rate of 10^10^ K/s, resulting in the Co_80_P_20_ metallic glass (MG) with a well-defined glass phase. NG model systems containing glassy grains with various sizes were constructed by filling glassy grains into a nanocrystalline model^53^, whose nanostructures such as grain sizes and shapes, interfaces, and triple junctions among grains could be well tuned to represent those observed in experiments. The procedures of construction of NG model systems were as follows: Firstly, atoms in Co_80_P_20_ MG model constructed by MD simulation as described above were filled into the regions defined as interiors of grains in the nanocrystalline model, forming NG models containing glassy grains with sizes varying from 3 to 32 nm. Secondly, adjacent glassy grains were prevented from being too close with each other, *i.e.*, the distances among those surface atoms across glassy grains were restricted to be larger than 1 nm, thereby forming NG models with well separated glassy grains (see Fig. 1). Subsequently, NG systems were kept at 500 K for 20 ns in MD simulation to equilibrate the free surfaces of glassy grains in the systems. Thirdly, NG model systems were compacted under the hydrostatic pressure of 1 bar, followed by subsequently cooling to 300 K; finally, the Co-P NG models containing 30–150 GGIs were obtained. Figure 1 demonstrates that the NG systems composed of glassy grains with various mean sizes, *i.e.*, *d* = 5, 7.5, 10, 20 nm, had been constructed for MD simulations, which were visualized by the OVITO software packages^54^.

## Results and discussion

### Materials characterization

**Figure 2:**
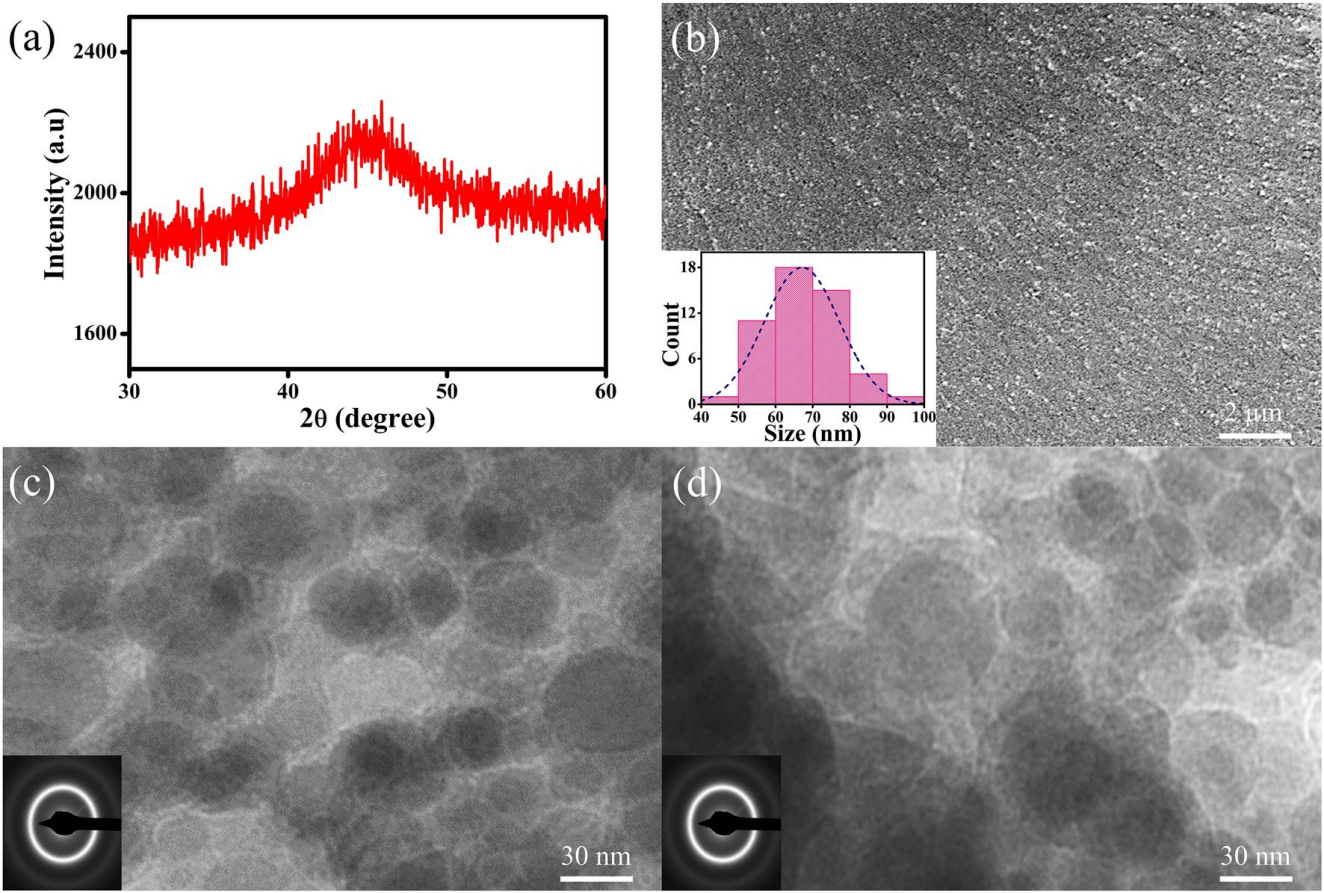
(**a**) XRD patterns, (**b**) SEM and (**c**,**d**) TEM images of NG films. The insets in (**b**–**d**) show the size distribution and SAED patterns, respectively.

**Figure 3:**
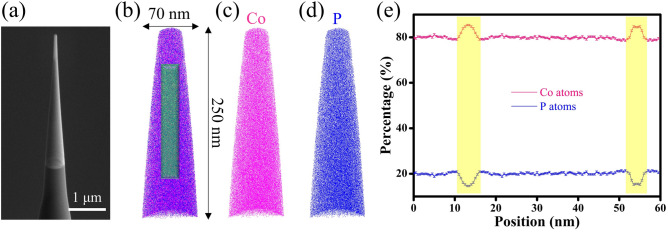
APT measurements on chemical composition of needle-shaped NGs (**a**) with elemental reconstruction of Co and P atoms (**b**–**d**). (**e**) The elemental concentrations are examined in the cylindrical region in (**b**). The highlighted areas in concentration profiles suggest the existence of two GGIs.

The XRD patterns of the free-standing Co-P films prepared by pulse electrodeposition are shown in Fig. 2a. A broad diffraction peak at 2θ ~ 45° is observed and no sharp diffraction peak associated with crystallization has been found, suggesting that the as-deposited specimens are fully amorphous. The SEM image illustrated in Fig. 2b demonstrates that the average size of glassy grains (D_avg_) is less than 100 nm, which is estimated to be D_avg_ = 67 ± 11 nm by calculating the mean diameter of at least 50 glassy grains, as shown in the size distribution (the inset in Fig. 2b). It is noteworthy that SEM image with a higher magnification is currently unavailable due to the magnetic nature of NG films. Figure 2c,d present the TEM images of NG films, showing that glassy grains with a diameter as small as 20 nm have been formed. A halo ring recognized from the selected area electron diffraction (SAED) pattern confirms the amorphous state of nanostructure. APT is further utilized to characterize needle-shaped NGs and the spatial distributions of Co and P elements are reconstructed at its tip, as demonstrated in Fig. 3a–d. Both types of atoms are well distributed throughout the examined regions, indicating that the GGIs do not contain any nanovoids. Nonetheless, an enrichment of Co atoms and a depletion of P atoms in an abnormal area of about 5 nm in width are evident (see Fig. 3e), which can be quantitatively described as Co_85_P_15_, as evaluated at the center of the GGI. In contrast, the glassy grains have a fairly homogeneous composition, *i.e.*, Co_80_P_20_. Such compositional heterogeneity suggests a *new glass phase* exists in Co-P NGs.

### Thermodynamic properties

**Figure 4:**
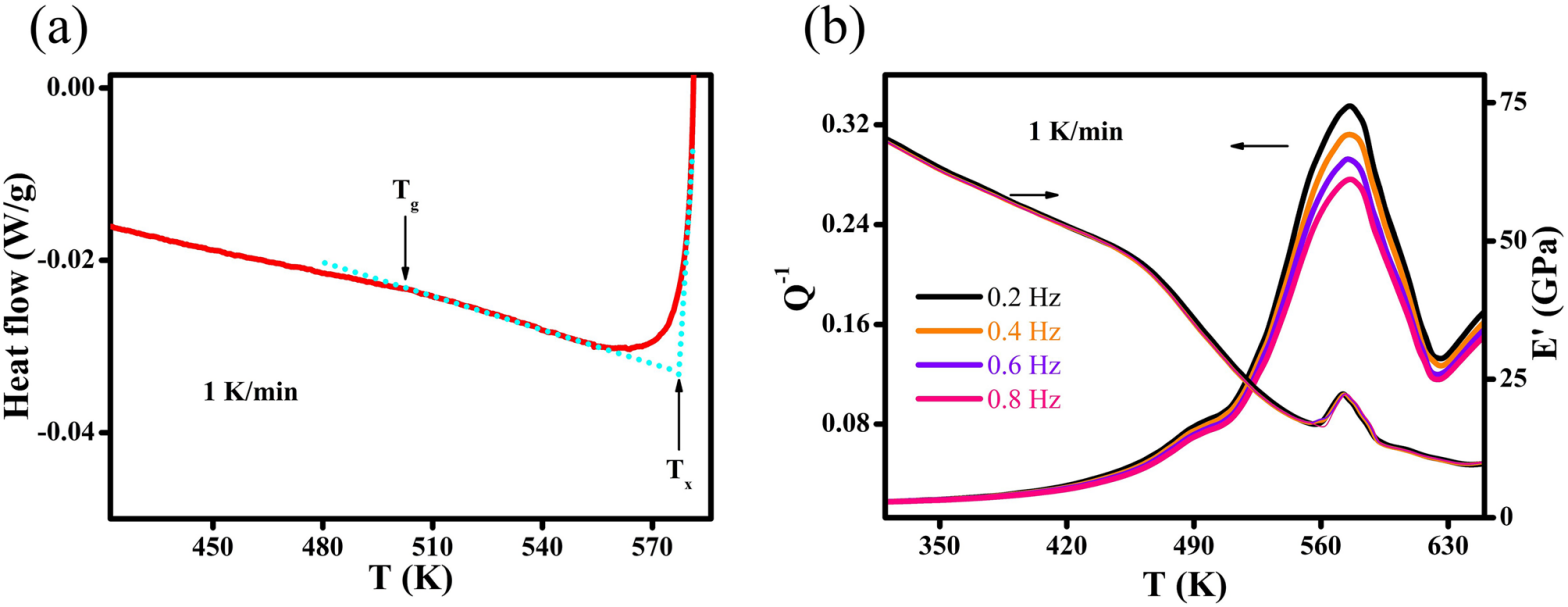
(**a**) Heat flows, (**b**) internal friction Q^−1^ and storage modulus E′ of NG films measured at a heating rate of 1 K/min. The dash line in (**a**) is tangent to the heat-flow curve, determining the onset temperatures of glass transition T_g_ and crystallization transformation T_x_.

Figure 4a presents the heat flows of NG films obtained from DSC measurements. Glass transition (T_g_ = 503 K) and crystallization transformation (T_x_ = 578 K) are identified at the onset temperatures of the endothermic and exothermic heat flow, as marked by arrows indicating the abrupt decrease and increase from lines tangent to the heat-flow curve, respectively. Moreover, the transition and evolution of nanostructures are evaluated by DMA, as shown in Fig. 4b. By elevating the temperature above 450 K, a dramatic reduction of storage modulus (E’) from 50 to 25 GPa is observed, resulting in an unapparent internal friction (Q^−1^) peak that slightly shifts to higher temperature as oscillating frequency increases from *f* = 0.2 Hz to 0.8 Hz. This Q^−1^ peak locates close to T_g_ measured by DSC. Therefore, the softening of nanostructures as caused by glass transition is evident. After transforming from the glass state into supercooled liquid state, a frequency-dependent Q^−1^ peak corresponding to the crystallization transformation is found to be prominent at T_x_, resulting in a transient increase and subsequent decrease of E’ above 560 K. The glass forming ability (GFA) of NG films may be well described by fitting crystallization Q^−1^ peak as follows^15^:

2 $${{\text{Q}}}^{-1}\left({{\text{T}}}_{{\text{x}}}\right)={{\text{T}}}_{{\text{x}}}\times {(\dot{{\text{T}}}/\upomega )}^{\upbeta },$$

where $\dot{{\text{T}}}$ is the heating rate, ω = 2π*f* is the angular frequency and Q^−1^(T_x_) is the internal friction at T_x_. In general, the Q^−1^ of amorphous alloys with an improved GFA would be more frequency dependent at an elevated temperature. The influences of GFA on the frequency-dependent Q^−1^ would reach the maximum at T_x_. Therefore, index β could be obtained by analyzing Q^−1^ at T_x_, *i.e.*, Q^−1^(T_x_). It is worth noting that the feasibility and sensitivity of index β obtained at T_x_ have been verified for MGs. Specifically, glassy alloys with a higher index β represents a better GFA and those with an index β > 0.4 are regarded to be good glass formers, which can be made into bulk MGs. Herein, index β = 0.14 is determined for NG films, substantially lower than the index β = 0.4. The compositional heterogeneity suggests that the GGIs are in a *new glass phase*, whose volume fraction x = 1 − [(D_avg_ − t)/D_avg_]^3^ is significant (x = 0.2) when D_avg_ = 67 nm for Co-P NGs, where t = 5 nm is the width of GGIs estimated by APT. In other words, glass phase of GGIs can be responsible for the small index β. Therefore, atomic structures of GGIs resulting from Co segregation could be in a thermodynamically unstable glass state, which would influence the mechanical properties of Co-P NGs.

### Mechanical properties

**Figure 5:**
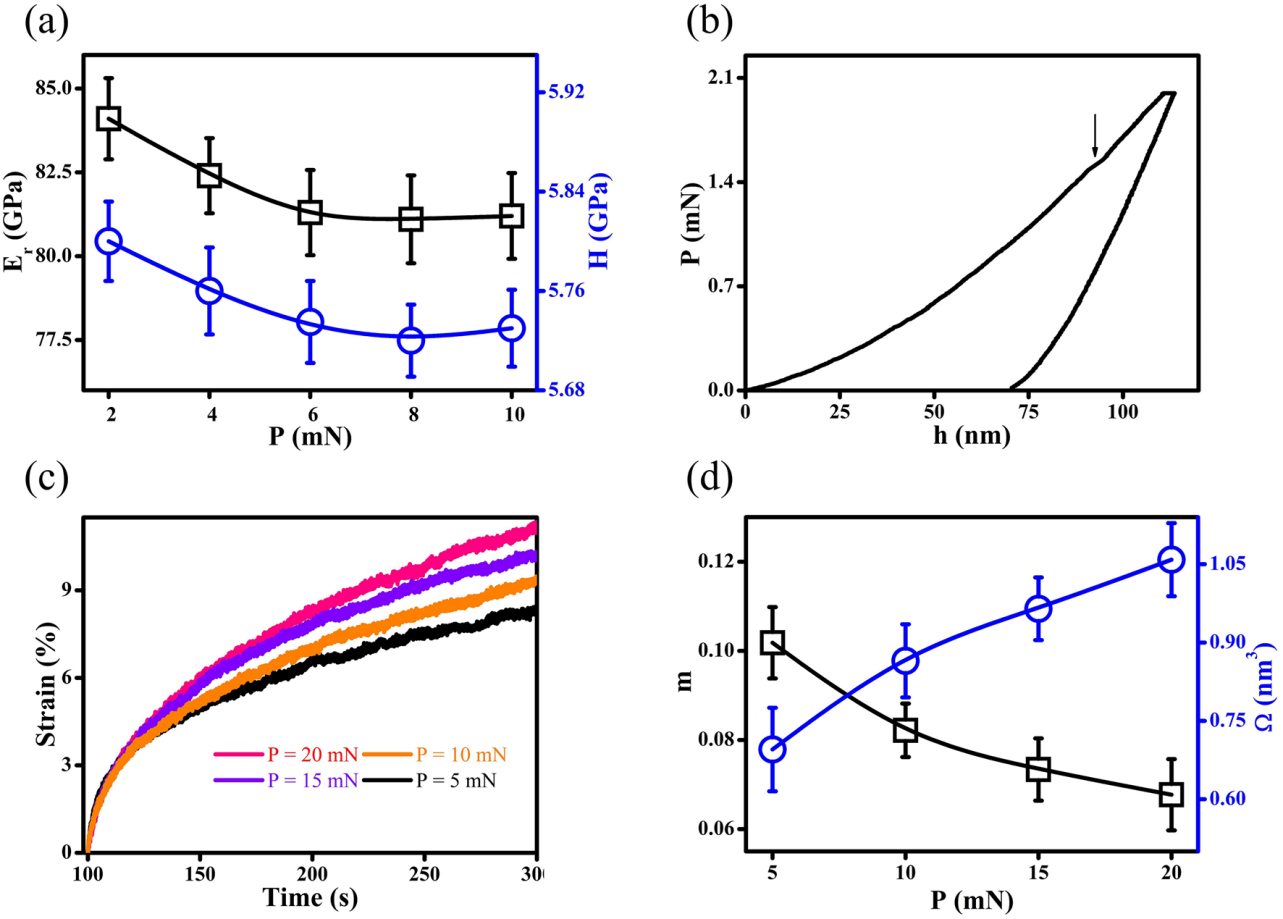
(**a**) Reduced modulus E_r_ and hardness H of NG films measured at the loading rate of 1 mN/s. (**b**) Load P vs. contact depth h plots; the arrow marks the first pop-in. (**c**) Creep curves, (**d**) SRS factor m and STZ volume $\Omega$ measured under different applied loads P.

Figure 5a presents the reduced modulus (E_r_) and hardness (H) evaluated under different applied loads (P) by nanoindentation measurements, suggesting that the NG films has E_r_ = 81.2 GPa and H = 5.73 GPa. A typical indentation curve with serration emerged at a contact depth (h = 90 nm) is shown in Fig. 5b, which could be correlated with compositional heterogeneity since it is close to D_avg_ = 67 nm of NG films. The appearance of serration in P–h plot is associated with shear banding, suggesting that the compositional heterogeneity would promote the shear banding under plastic deformation. The creep curves illustrated in Fig. 5c are fitted using an empirical equation ${\text{h}}\left({\text{t}}\right)={{\text{h}}}_{{\text{o}}}+{\text{a}}{({\text{t}}-{{\text{t}}}_{{\text{o}}})}^{{\text{b}}}+{\text{kt}}$, where h_o_ is the initial contact depth, t_o_ is the initial time, and a, b, and k are fitting constants. The strain rate in creep is determined at the steady state (${\dot{\upepsilon }}_{{\text{ss}}}$) and then used to estimate strain rate sensitivity (SRS) factor m as follows:

3 $${\text{m}}=\frac{\partial {\text{ln}}\left(\upsigma \right)}{{\partial }{\text{ln}}\left({\dot{\upepsilon }}_{{\text{ss}}}\right)},$$

where σ = P/24.5h^2^ is applied stress. Shear transformation zone (STZ) volume $\Omega$ is evaluated based on the models developed by Johnson et al.^55^ and Pan et al.^56^ as follows:

4 $$\Omega =\frac{{{\text{k}}}_{{\text{B}}}{\text{T}}}{{{\text{C}}}^{{^{\prime}}}{\text{mH}}},$$

where k_B_ is the Boltzmann constant, T is the temperature, ${{\text{C}}}^{{^{\prime}}}=2{{\text{G}}}_{0}\mathrm{R\zeta }{\upgamma }_{{\text{c}}}^{2}\sqrt{1-{\uptau }_{{\text{CT}}}/{\uptau }_{{\text{c}}}}/\sqrt{3}{\uptau }_{{\text{c}}}$, ${{\text{G}}}_{0}$ is the shear modulus at 0 K, R ≈ 0.25 and $\upzeta$ ≈ 3 are the constants, ${\upgamma }_{{\text{c}}}=0.027$ is the average elastic strain limit, ${\uptau }_{{\text{CT}}}$ is the threshold shear resistance at the temperature T, ${\uptau }_{{\text{c}}}$ is the threshold shear resistance at 0 K, ${{\text{G}}}_{0}/{\uptau }_{{\text{c}}}\approx 27.78$, and ${\uptau }_{{\text{CT}}}/{\uptau }_{{\text{c}}}=1-(0.016/0.036){({\text{T}}/{{\text{T}}}_{{\text{g}}})}^{2/3}$. In general, glassy alloys with a higher SRS factor m and a lower STZ volume $\Omega$ are expected to better resolve the issue of brittleness, thereby preventing brittle fractures or even achieving super-plasticity. When P has been increased, SRS factor m and STZ volume $\Omega$ are found to decrease and increase, respectively (see Fig. 5d). Specifically, at P = 5 mN, m = 0.10 and $\Omega$=0.69 nm^3^ are determined for the NG films with D_avg_ = 67 nm, whose ductility might be further improved. To be specific, it is speculated that Co-P NGs with increasing compositional heterogeneity, namely, increasing number of Co atoms segregated to GGI regions, would have decent improvements in ductility. Such speculation could be further studied by MD simulations.

### MD simulations

**Figure 6:**
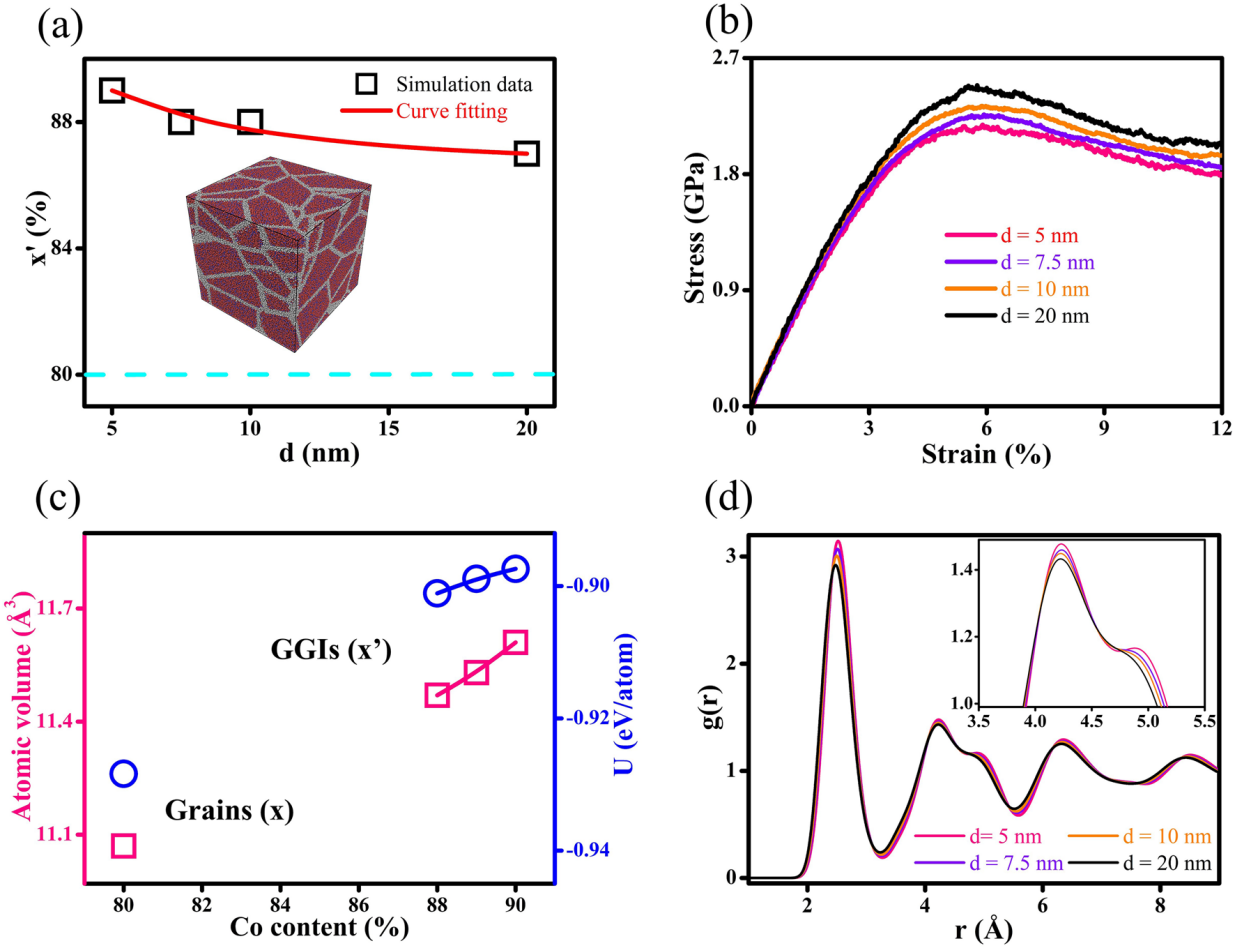
(**a**) The compositions of GGIs (x’, black symbols) versus *d*; the dash line represents that (x = 80) of interiors of glassy grains. The inset shows the Co-P NGs in MD simulations. (**b**) The stress versus strain curves of Co-P NGs with different *d*. (**c**) The atomic volume and atomic internal energy U of GGIs with Co contents of x’ = 89, 88 and 87, and interiors of glassy grains with a Co content of x = 80. (**d**) The influences of *d* on the RDFs of Co-P NGs; inset: enlarged view on the second RDF peak.

Atomistic models of nanostructures containing glassy grains are constructed to simulate Co–P NGs synthesized through the pulse electrodeposition (the inset in Fig. 6a), which are employed to explore the influences of compositional heterogeneity on mechanical properties of Co-P NGs. These NG systems with *d* = 5, 7.5, 10 and 20 nm contain two distinct glassy phases, *i.e.*, GGI regions with x’ and interiors of glassy grains with x = 80. Herein, x is the Co content in the interiors of glassy grains while x’ is defined as the average Co content in the GGI regions. Figure 6a shows that there is an increase in x’ when *d* gets smaller in Co-P NGs, *i.e.*, Co content x’ varies from x´ = 88 to 90 when *d* reduces from *d* = 20 nm to 5 nm, manifesting the fact that the compositional heterogeneity is grain-size dependent and it could be enhanced in Co-P NGs with a lower *d*.

**Figure 7:**
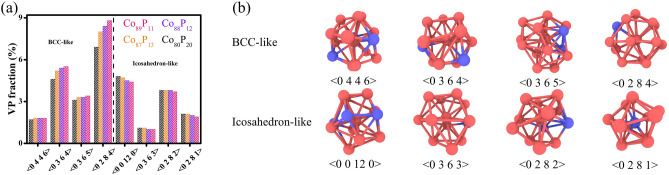
(**a**) The VPs of GGIs with various Co contents (x’ = 89, 88 and 87) and interiors of Co_80_P_20_ glassy grains. (**b**) Schematics of bcc- and icosahedron-like VPs.

The stress vs. strain curves shown in Fig. 6b are determined by MD simulations on the NG systems. If *d* is reduced from 20 to 5 nm, the mechanical strength of NG systems would decrease monotonously, which can be attributed to the increasing compositional heterogeneity resulting from the decreasing *d* since the mechanical strength of GGIs could be reduced by an increasing Co content x’. Figure 6c demonstrates that the GGIs with Co segregation are less dense than the interiors of glassy grains (with x = 80) and there is an increase in atomic volume once if Co content x’ is increased, meaning that the excess free volumes would be created at interfaces by Co segregation. The composition-dependent atomic internal energy (U) illustrated in Fig. 6c suggests that the *new glass phase* resulting from Co segregation at the GGIs is in a much higher energy state, as compared to the interiors of glassy grains. The segregation of Co atoms to the GGI regions may be well reflected by the analyses on the RDFs of NG systems shown in Fig. 6d. Obviously, the peak intensities increase with decreasing *d*. Particularly at the shoulder of the second peak, the enhanced intensity is clearly an indication on the formation of a *new glass phase*. Considering the increasing compositional heterogeneity with decreasing *d*, atomic structures of GGIs with an increased Co content x’ are thus suggested to much differ from those at the interiors of glassy grains. Such feature is further investigated by analyzing the Voronoi polyhedrons (VPs) in the GGI regions (x’) and in the interiors of Co_80_P_20_ glassy grain, as shown in Fig. 7a. The Voronoi index, < n_3_ n_4_ n_5_ n_6_ > , is employed to identify a VP consisting of n_3_, n_4_, n_5_, and n_6_ number of faces with 3, 4, 5, and 6 edges, respectively. It is worth noting that the bcc-like and icosahedron-like VPs would possess a coordination number (n_3_ + n_4_ + n_5_ + n_6_) of 13–14 and 11–12, respectively, which are all dominant in the overall population of VPs, as visualized in Fig. 7b. The fractions of bcc-like VPs at GGI regions with a higher Co content x’ are more significant, particularly for the < 0 2 8 4 > VPs. Therefore, it may be concluded that the enhanced peak intensity with decreasing *d* observed from the RDF analysis is attributed to the increase in the fraction of bcc-like VPs and the glass phase of GGIs containing more bcc-like VPs could be less disordered than that in the interiors of glassy grains. In contrast, the fractions of icosahedron-like VPs at the GGIs with a higher Co content x’ have been reduced, especially for those characterized as the < 0 0 12 0 > . In general, glassy alloys containing more icosahedron-like VPs would have larger shear resistance and exhibit higher mechanical strength, which are harder to be deformed by externally applied loads. Thus, mechanical strength of Co-P NGs decreases through enhancing compositional heterogeneity. Additionally, compositional heterogeneity could be beneficial to promoting the shear banding in NGs, simply because the shear resistance of GGIs would be reduced with increasing x’.

**Figure 8:**
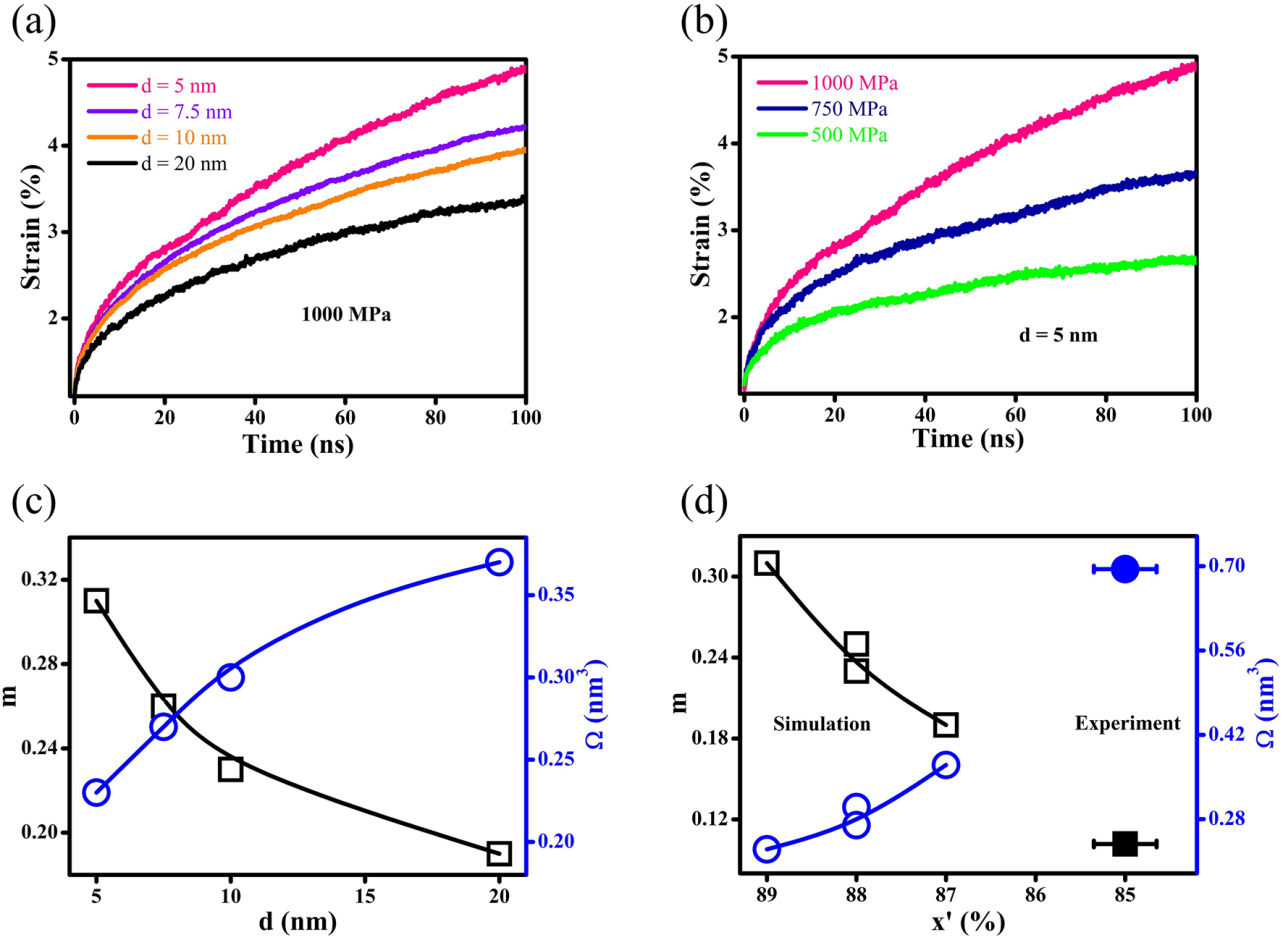
The influences of (**a**) *d* and (**b**) σ on creeps of NG systems. (**c**) Size effects on SRS factor m and STZ volume $\Omega$, as determined from MD simulations; the lines are the guides to the eye. (**d**) The influences of GGI compositions on SRS factor m and STZ volume $\Omega$, as evaluated by MD simulations (hollow symbols) and nanoindentation (solid symbols); the lines are the guides to the eye.

The creep deformations of NG systems under an applied stress of 1000 MPa are illustrated in Fig. 8a. For a given time (100 ns), Co-P NGs with a reduced *d* achieve a much-enhanced strain rate in creep, which could be attributed to the increase in the compositional heterogeneity. Similarly, the SRS factors m of Co-P NG model systems can be determined by fitting Eq. (3) with the strain rate in creep obtained at the steady state under σ = 500, 750 and 1000 MPa, as shown in Fig. 8b. Figure 8c demonstrates that there are increases in the SRS factor m and decreases in the STZ volume $\Omega$ when *d* is reduced, suggesting that the enhanced compositional heterogeneity resulting from a higher Co content x’ of GGIs can improve the ductility of NGs. It may be explained by the different roles of GGIs enabled through the different number of Co atoms segregated to the GGIs. For GGIs with reduced Co segregation, the shear resistance of GGIs can be similar with those in the interiors of glassy grains or MGs. Thus, the shear banding in Co-P NGs would be analogous to that of MGs without GGIs, whose plastic deformation is inhomogeneous and the fracture is brittle. In contrast, the number of shear bands formed at the GGIs with a higher Co content x’ can be prominent due to the lower shear resistance. Clearly, the global existence of GGIs with enhanced Co segregation might lead to homogenous plastic deformation since the flows of activated defects would slide into other GGIs, resulting in decent improvements in the ductility. Therefore, it is the compositional heterogeneity that improves the ductility of Co-P NGs, as reflected by the SRS factor m and STZ volume $\Omega$ dependences on the Co content x’ shown in the Fig. 8d, which have been evaluated from MD simulation and nanoindentation experiments.

## Conclusion

In summary, Co-P NGs are systematically studied and the influences of compositional heterogeneity on the mechanical properties of Co-P NGs have been analyzed in detail. The compositional heterogeneity as described by the chemical composition of GGIs are evaluated by APT measurements and MD simulations, which well reflects the segregation of alloying element Co to the GGI regions. It is suggested that there exists a *new glass phase* in the GGI regions, which can be less dense than that in the interiors of glassy grains, as caused by Co segregation. Such compositional heterogeneity is beneficial to the improvements in ductility of Co-P NGs and would render Co-P NGs with a better capability to resolve the issues of their intrinsic brittleness.

## Data Availability

The datasets generated and/or analyzed during the current study are available from the corresponding author on reasonable request.
